# Strain and Temperature Sensitivities Along with Mechanical Properties of CNT Buckypaper Sensors

**DOI:** 10.3390/s20113067

**Published:** 2020-05-28

**Authors:** Shiuh-Chuan Her, Wei-Chun Hsu

**Affiliations:** Yuan Ze University, Chung-Li 320, Taiwan; s1045026@mail.yzu.edu.tw

**Keywords:** buckypaper, strain sensitivity, temperature sensitivity, thermal stability

## Abstract

In this work, buckypaper composed of multi-walled carbon nanotubes (MWCNT) was prepared through a vacuum filtration process. The effect of MWCNT aspect ratio on the buckypaper performance was investigated. The freestanding and highly flexible buckypaper can be used as a sensor to attach on a complex surface monitoring the strain and temperature at the critical area. The mechanical properties of the buckypaper were examined using the tensile and nanoindentation tests. The strain and temperature sensitivities of the buckypaper were evaluated through the four-point bending and thermal chamber tests, respectively. In addition, the microstructure and thermal stability of the buckypaper were studied by scanning electron microscopy (SEM) and thermogravimetric analyzer (TGA), respectively. Experimental results showed that the mechanical properties such as Young’s modulus, tensile strength, fracture strain, and hardness of the buckypaper made of high aspect ratio MWCNTs were significantly superior to the buckypaper consisted of low aspect ratio MWCNTs, while the strain and temperature sensitivities of the buckypaper composed of low aspect ratio MWCNTs were better than that of the buckypaper made of high aspect ratio MWCNTs.

## 1. Introduction

Since the discovery in 1991 by Iijima [1], carbon nanotubes (CNTs) have received great attention in a variety of research fields owing to their extraordinary mechanical, thermal, and electrical properties. Pipe et al. [2] predicted the Young’s modulus of CNT in the range of 500 ~ 600 GPa based on both the theoretical analysis and experimental results. Yu et al. [3] reported that the maximum tensile strength of CNT is approximate to 200 GPa. Yang et al. [4] indicated that the thermal conductivity of a randomly oriented multi-walled carbon nanotube (MWCNT) film is close to 15 W/mK. Gao et al. [5] found that the electrical resistivity of CNT is less than $10^{-5}$ Ω/cm. CNTs with a great flexibility compared with conventional carbon materials have been considered as one of the most promising materials for high performance of structural and multifunctional applications. Despite their remarkable features, limitations in terms of handling and operating of the nanomaterial have significantly restricted the potential usage of CNTs. Thus, many applications can only be realized by developing alternate approaches that can take the advantages of the unique property of individual CNT and translate into macroscopic scale. One of the most important approach in this subject is the preparation of CNT film. The flexible and free-standing CNT film known as buckypaper is a macroscopic paper-like material with planner structure comprising of porous and entangled network of CNTs formed by Van der Waals forces [6].

Buckypaper with high electric and thermal conductivities [7] has attracted a great attention for various applications in recent years. Potential applications of buckypaper reported in the literatures including transistors and logic devices [8], supercapacitors [9], actuators [10], and strain sensing [11]. Lu et al. [12] fabricated the buckypaper using spray-vacuum filtration method to monitor the glass transition temperature of polymeric composites. Zhang et al. [13] investigated the structural, mechanical, and conductive properties of buckypaper prepared by pressurized filtration method. Wang et al. [14] studied the piezoresistive behavior of CNT composite film subjected to tensile and laterally compressive loads. Zhang et al. [15] improved the mechanical and electrical properties of buckypaper by in situ cross-linking of CNTs. Lu et al. [16] monitored the curing behavior of epoxy/glass composite using highly sensitive buckypaper. Arif et al. [17] demonstrated that both the electrical and thermal conductivities of compressed buckypaper are increasing due to the higher density of CNT network. Li et al. [18] investigated the influence of CNTs synthesized over different catalysts on the performance of buckypaper. Wang et al. [19] fabricated the buckypaper using spray vacuum filtration method and embedded into glass fiber reinforced polymer composite laminate to monitor the drilling process. Li et al. [20] embedded buckypaper into glass fiber reinforced plastics laminates to enhance the interlaminar fracture toughness and shear strength. Dao et al. [21] developed Limnobium Laevigatum nanogenerator (LLN) for electricity generation and freshwater production using vacuum filtration deposition of MWCNTs onto cellulose paper-located on the Polystyrene (PS) foam. CNTs served as sunlight absorbers have been widely used in solar-driven steam generation devices with a variety of applications such as low-cost steam for medical sanitization, seawater desalination, chemical distillation and water purification [22,23,24]. Lu et al. [25] developed a flexible spongy CNTs consisting of self-assembled, interconnected CNT skeletons, was directly used as electromagnetic interference (EMI) shielding film. The freestanding CNT sponge with a thickness of 1.8 mm showed highly EMI shielding effectiveness (SE) and specific SE (SSE) of 54.8 dB and 5480 dB $cm^{3}/g$ in X-band, respectively. Dao and Choi [26] used dry plasma reduction (DPR) to synthesis MWCNT–Platinum nanohybrid acted as an efficient and low-cost counter electrode material for dye-sensitized solar cells. Dang et al. [27] prepared CNT-Ruthenium nanohybrid using liquid plasma reduction for the redox reaction in dye-sensitized solar cells. Kim et al. [28] prepared a hybrid electrode based on sulfur-impregnated MWCNTs using a dry plasma reduction method for lithium–sulfur batteries. The MWCNT-SnO electrode exhibited high conductivity and small volume change during a repeated charge–discharge process.

The basic concept behind the preparation of buckypaper is to utilize the exceptional properties of CNTs in macroscopic scale. The macroscale buckypaper can be handled easily and improve the feasibility of using CNTs in industries. It has been reported that the performance of buckypaper is significantly affected by several important factors such as CNTs type [29], aspect ratio of CNTs [30], degree of CNT alignment [31], chemical functionalization of CNT [32], and purity [33]. In this work, the buckypaper composed of MWCNTs was prepared through a vacuum filtration process. The mechanical properties including the Young’s modulus, tensile strength, fracture strain, and hardness were examined using the tensile and nanoindentation tests. Thermal stability of the buckypaper was evaluated by thermogravimetric analyzer (TGA). Buckypaper with remarkable electrical and thermal conductivities has been considered as an ideal sensor for strain and temperature sensing. The sensitivities of strain and temperature were investigated through a series of four-point bending and thermal chamber tests, respectively. The effect of MWCNT aspect ratio on the mechanical properties and sensing capability was studied.

## 2. Experimental

### 2.1. Preparation of Buckypaper

**Figure 1:**
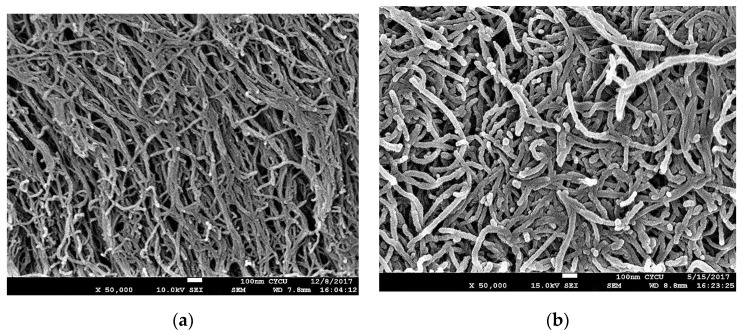
Morphologies of multi-walled carbon nanotubes (MWCNTs). (**a**) high aspect ratio MWCNTs, (**b**) low aspect ratio MWCNTs.

**Figure 2:**
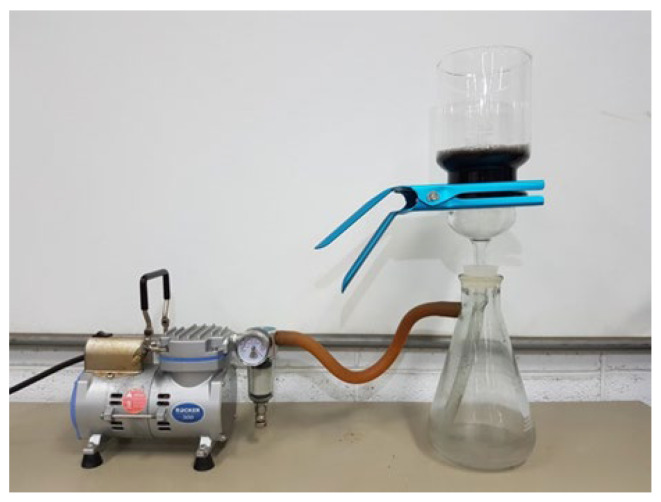
Experimental setup of vacuum filtration.

**Figure 3:**
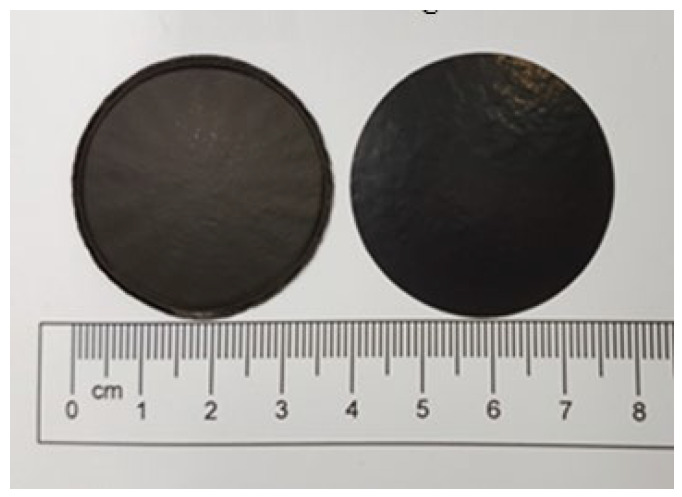
Buckypapers as prepared.

**Figure 4:**
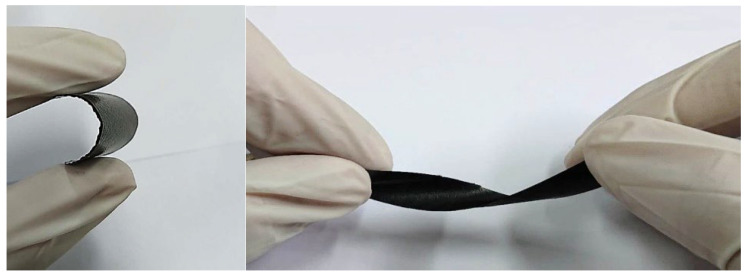
Bending and twisting tests of Buckypapers.

Two different sizes of MWCNTs were used in this work to investigate the influence of the MWCNT aspect ratio on the buckypaper performance. For the high aspect ratio MWCNTs, the diameter and length are ranging from 10–20 nm and 100–200 μm, respectively. For the low aspect ratio MWCNTs, the diameter and length are ranging from 30–50 nm and 50–100 µm, respectively. In this work, MWCNTs were purchased from Conjutek Co., New Taipei City, Taiwan, and used as received without any further purification. The purities of high aspect ratio MWCNTs and low aspect ratio MWCNTs were ≥98.5% and ≥97%, respectively. Figure 1 shows the morphologies of the MWCNTs as received. The buckypaper was fabricated using vacuum filtration method with the aid of surfactant Triton X-100 (Big Sun Chemical Corp., New Taipei City, Taiwan). The vacuum pressure used in the filtration process was 0.1 atm. Triton X-100 (C_14_H_22_O(C_2_H_4_O)*_n_*) purchased from Big Sun Chemical Corp., New Taipei City, Taiwan, which is a nonionic surfactant with a hydrophilic polyethylene oxide chain. It has a viscosity of 400 centipoise at 25 °C and purity of 97% and is soluble in water. To overcome the van der Waals forces between MWCNTs and break up MWCNTs bundles into individual MWCNTs, a series of experimental tests were conducted to obtain the optimal preparation process. The optimal preparation processes were as follows: 50 mg of MWCNTs and 5 g of Triton X-100 were added into 250 mL distilled water. Then, the mixture was dispersed by a sonicate tip at 30 W for 2 h. The sonicator (Q700, Qsonica L.L.C., Newtown, CT, USA) was operated at pulse mode with 10 s on and 20 s off. Upon the completion of the dispersion procedure, the well dispersed suspension was filtered through a PVDF (Polyvinylidene Fluoride) microporous membrane with pore size 0.45 μm and diameter 47 mm. Figure 2 shows the experimental setup of the vacuum filtration. After the filtration, the buckypaper was peeled off from the PVDF membrane and soaked in the isopropyl alcohol for 4 h to remove the residual surfactant Triton X-100. Then, the buckypaper was washed by a total amount of 5 L DI water and dried in a thermal chamber at the temperature of 40 °C for 5 h. A typical free standing and flexible buckypaper is illustrated in Figure 3. The diameter and thickness of buckypapers were 36 mm and 0.1 mm, respectively. The thickness of the buckypaper was measured by micrometer caliper (Mitutoyo Taiwan Co., Ltd.) with an accuracy of 0.001 mm. It can be seen from the naked eye that the fabricated buckypaper exhibits smooth surface with black color and no crack on the surface. Buckypaper has good strength and flexibility to be handled like a traditional fiber mat. Bending and twisting tests were conducted on the buckypaper as shown in Figure 4 to illustrate the high flexibility of the free standing buckypaper.

### 2.2. Testing and Characterization

The surface morphology of the buckypaper was examined by a field emission scanning electron microscopy (JSM 7600F, Jeol Co. Japan) operated at an accelerated voltage of 10 kV. The test sample was coated with platinum. The thermal stability of the buckypaper was tested with a thermogravimetric analyzer (TGA) (2-HT TGA, Mettler Toledo, USA). The test sample was placed in a thermal chamber filled with nitrogen. The temperature was increased from room temperature to 800 °C at a rate of 10 °C/min. In this work, mechanical properties of the buckypaper such as Young’s modulus, tensile strength and fracture strain were characterized using the tensile test while the hardness was determined by nanoindentation test. Tensile tests for the buckypaper were conducted on a strip sample (30 mm × 10 mm) using a universal tensile machine operated at a constant speed of 0.5 mm/min. To avoid the stress concentration at the grip, a rubber pad was attached to the grip. Nanoindentation tests were performed using a Nano indenter (NanoTestⅠMicro Materials Ltd., UK) equipped with a Berkovich diamond indenter to evaluate the hardness of the buckypaper.

To investigate the strain sensitivity, a buckypaper sensor with dimensions of 30 mm × 10 mm was bonded on an aluminum test specimen using epoxy adhesive and subjected to four-point bending. The resistance change of the buckypaper due to the applied strain was measured by a source meter (Keithley 2450, USA). The strain sensitivity of the buckypaper is quantified as the relative resistance change owing to the applied strain, commonly referred to gauge factor as follows.

(1) $$\mathrm{GF}=\frac{△R}{R_{0}\varepsilon }$$

where $R_{0}$ and ∆R are the initial resistance and relative resistance change due to the applied strain ε, respectively.

To evaluate the temperature sensitivity, the buckypaper sensor was put into a vacuum oven and temperature varied from room temperature to 100 °C. A K-type thermocouple was placed next to the buckypaper to measure the temperature. The resistance change of the buckypaper sensor was measured by a Keithley 2450 source meter. Temperature coefficient of resistance (TCR) was used to characterize the temperature sensitivity as follows.

(2) $$\mathrm{TCR}=\frac{∆R}{R_{0}∆T}$$

where $R_{0}$ is the initial resistance at room temperature, ∆R is the relative resistance change due to the temperature change ∆T.

## 3. Results and Discussion

### 3.1. Morphology of Buckypaper

**Figure 5:**
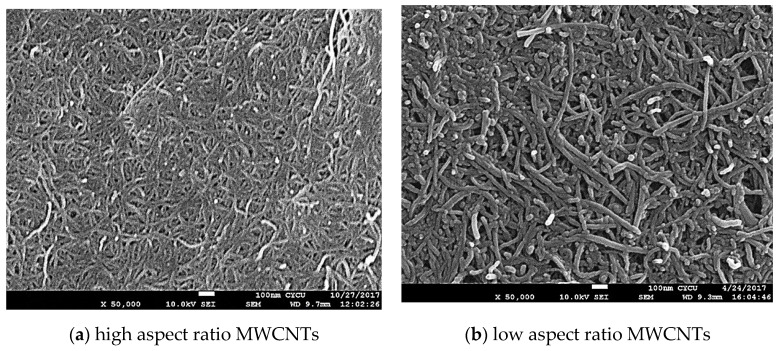
Surface morphology of buckypaper. (**a**) buckypaper composed of high aspect ratio MWCNTs (**b**) buckypaper composed of low aspect ratio MWCNTs.

**Figure 6:**
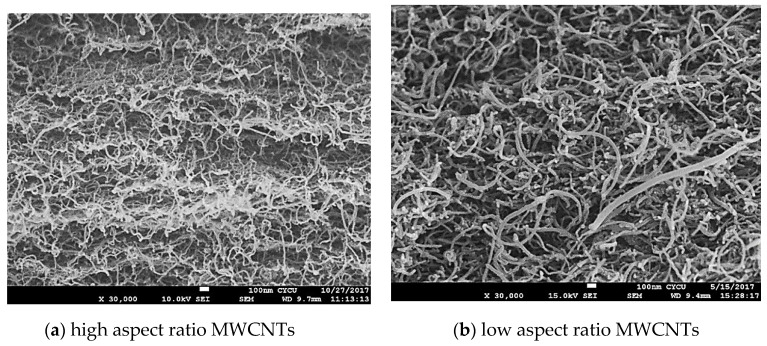
Cross section of buckypaper: (**a**) buckypaper composed of high aspect ratio MWCNTs and (**b**) buckypaper composed of low aspect ratio MWCNTs.

Figure 5 shows the surface morphologies of buckypapers fabricated from high and low aspect ratios MWCNTs. It appears that the entangled MWCNT network consists of continuous MWCNT ropes resulting from the self-assembly due to the van der Waal forces. The well distributed network, uniform rope size, and porous structure of the buckypaper demonstrate a good dispersion of MWCNTs in the suspension. The buckypaper with high aspect ratio WMCNTs forms more porous network. As MWCNTs pile up, the gap between MWCNTs is increasing with the increase of the MWCNT diameter. Thus, the pore size of the buckypaper (piled up of MWCNTs) with low aspect ratio MWCNTs is larger than that of high aspect ratio MWCNTs. It can be seen from the surface morphology as shown in Figure 5 that the pore size and the rope diameter of the entangled network are dependent on the MWCNT diameter, the larger of the MWCNT diameter the larger of the pore size and rope diameter. Figure 6 shows the cross section of the buckypapers. The test sample used for the cross-section SEM image was obtained from the broken part of the tensile test specimen. A layered structure was observed in the buckypaper made of high aspect ratio MWCNTs. While a loose interlayer structure was found in the buckypaper composed of low aspect ratio MWCNTs due to the less tightly bound between the low aspect ratio MWCNTs. The bonds between MWCNTs were evaluated based on the degree of entanglement. MWCNTs with high aspect ratio exhibit a higher degree of entanglement in comparison with low aspect ratio MWCNTs. Thus, a high degree of entanglement leads to tight bonds between MWCNTs with a high aspect ratio. This indicates that a better interconnection between the high aspect ratio MWCNTs in comparison with low aspect ratio MWCNTs.

### 3.2. Thermal Stability

**Figure 7:**
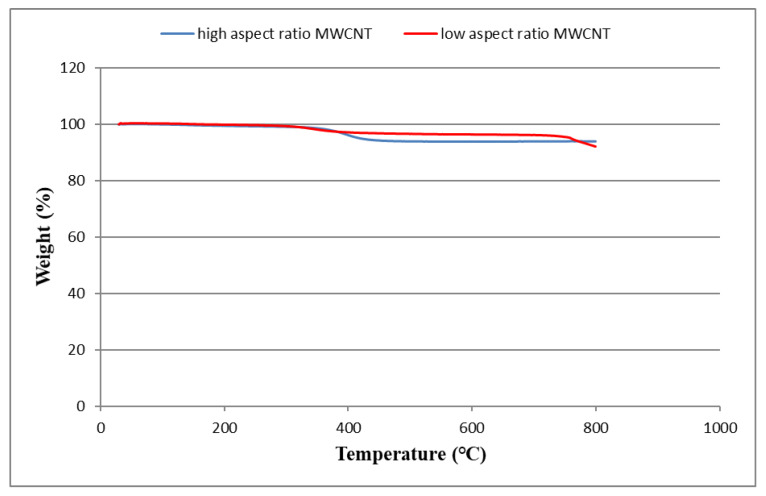
Residual weights of buckypapers composed of high and low aspect ratios MWCNTs in the thermogravimetric analyzer (TGA) test.

Figure 7 shows the residual weight of the buckypaper as the temperature increasing from room temperature to 800 °C. The residual weight percentages of buckypapers composed of high aspect ratio MWCNTs and low aspect ratio MWCNTs at the temperature of 800 °C are 93.5% and 91.9%, respectively. It can be observed that a good thermal stability was achieved for the fabricated buckypapers. Most of the weight loss can be attributed to the residual surfactant Triton X-100 or moisture in the buckypaper.

### 3.3. Mechanical Properties

**Figure 8:**
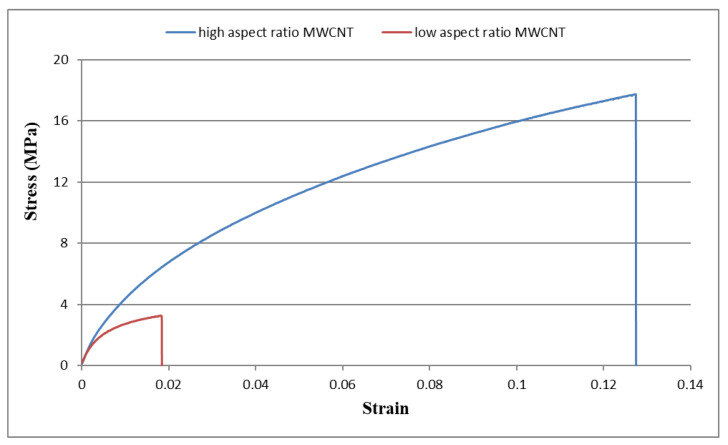
Stress–strain curves of buckypapers composed of high aspect ratio MWCNTs and low aspect ratio MWCNTs.

Typical stress–strain curves for the buckypapers made of high and low aspect ratios of MWCNTs are presented in Figure 8. The Young’s modulus, tensile strength, and fracture strain of the buckypaper can be extracted from the stress–strain curve. Three samples were prepared and tested for each buckypaper. The average values and standard deviations of the tensile properties of the buckypaper are listed in Table 1. It is clear that all the tensile properties of the buckypaper composed of high aspect ratio MWCNTs are much higher than that of the buckypaper made of low aspect ratio MWCNTs. This demonstrates that the reinforcement of MWCNTs with large diameter is significantly deteriorated in comparison with small MWCNTs. Sastry et al. [34] pointed out that the tensile properties of porous fiber materials are heavily dependent on the load transfer ability among the networks. The buckypaper with high aspect ratio WMCNTs forms a more porous network. As MWCNTs pile up, the gap between MWCNTs is increasing with the increase of the MWCNT diameter. Thus, the pore size and rope diameter of the buckypaper composed of high aspect ratio MWCNTs are smaller than that of the buckypaper consisted of low aspect ratio MWCNTs as shown in Figure 5. Buckypaper made of high aspect ratio MWCNTs exhibits a denser pack compared with buckypaper consisted of low aspect ratio MWCNTs. The larger pore size and rope diameter decrease the load transfer between tube/tube in the MWCNT rope due to the possible tube slippage. In addition, the layered structure of the buckypaper composed of high aspect ratio MWCNTs as shown in Figure 6a indicates a strong interconnection between the MWCNT ropes resulting in a good load transfer among the MWCNT networks. The interconnection between MWCNTs is attributed to the entanglement of MWCNTs network. The degree of entanglement of MWCNTs with high aspect ratio is higher than that of low aspect ratio MWCNTs. The better tensile properties of the buckypaper composed of high aspect ratio MWCNTs demonstrate that the interconnection between the high aspect ratio MWCNTs is better than that of low aspect ratio MWCNTs.

**Figure 9:**
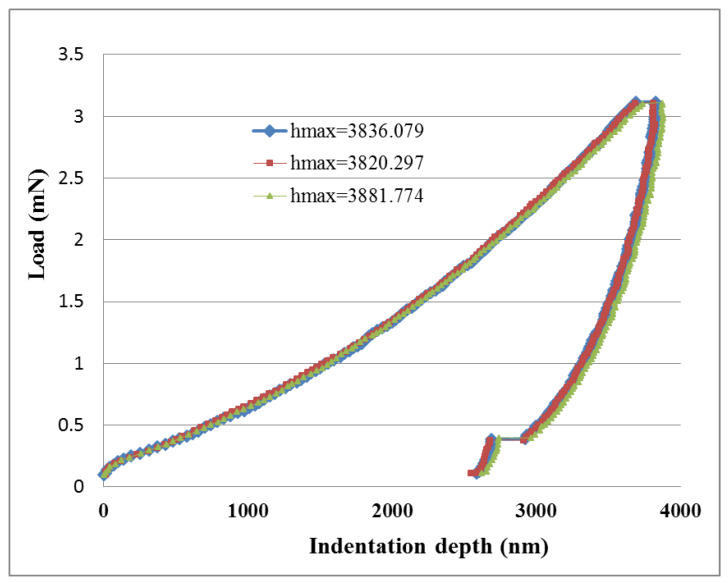
Load vs. indentation depth for the buckypaper made of high aspect ratio MWCNTs.

**Figure 10:**
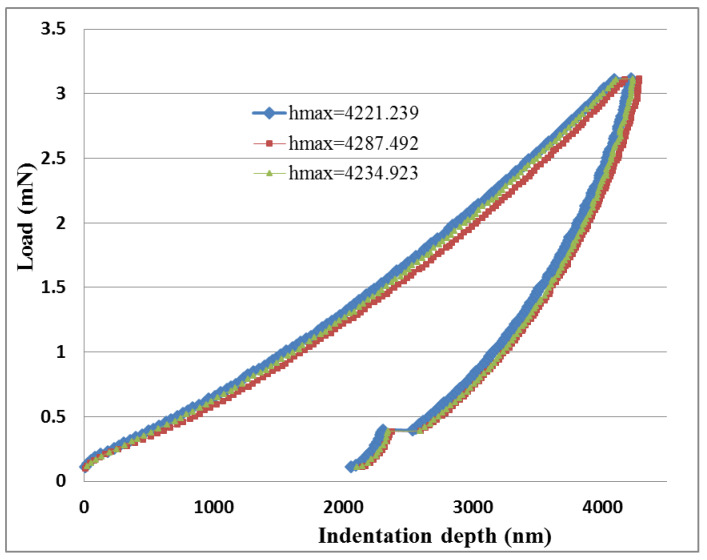
Load vs. indentation depth for the buckypaper made of low aspect ratio MWCNTs.

In the nanoindentation tests, the maximum indentation load used was 3 mN. Three nanoindentation tests were conducted for each test sample. Figure 9 and Figure 10 plot the load versus indentation depth curves for high aspect ratio MWCNTs and low aspect ratio MWCNTs buckypapers, respectively. Three nanoindentation tests were conducted on each buckypaper to illustrate the repeatability. The hardness of the buckypaper can be extracted from the load-indentation depth curve using the expressions proposed by Oliver and Pharr [35]. The hardness of the buckypapers composed of high aspect ratio MWCNTs and low aspect ratio MWCNTs are 112 ± 2 MPa and 107 ± 1 MPa, respectively.

### 3.4. Strain Sensitivity

**Figure 11:**
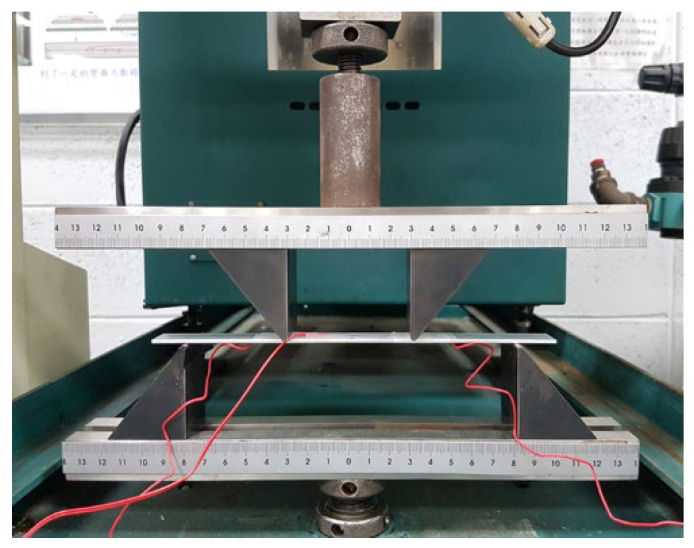
Experimental setup of the four-point bending test.

The prepared buckypaper was cut into rectangular strips with dimensions of 30 mm × 10 mm as a sensor for evaluating the piezoresistive response and sensing performance. An aluminum beam with dimensions of 200 × 20 × 2 mm was used as a test specimen. The buckypaper sensor and strain gauge were adhered to the top and bottom surfaces of the Al beam in the central region, respectively. The Al specimen was subjected to four-point bending test as shown in Figure 11. The distances between the two inner points and two outer points for the four-point bending test were 60 mm and 160 mm, respectively. The specimen was loaded by a tensile machine (10 KS, Hounsfield, United Kingdom) at a loading speed of 5 mm/min. The electrical resistance of the buckypaper was measured by a source meter (Keithley 2450, Tektronix, Inc., Beaverton, OR, USA). A DC voltage was applied then the resistance of the buckypaper can be calculated from the applied voltage and measured current based on the Ohmic law. Silver paste was used as electrodes to minimize the contact resistance. The change of the resistance of the buckypaper was measured as a function of the strain applied to the specimen.

**Figure 12:**
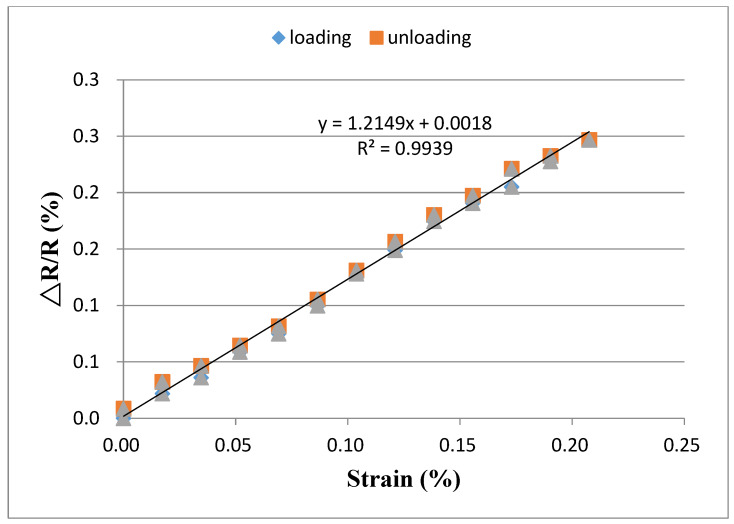
Relative resistance change vs. applied strain for the buckypaper made of high aspect ratio MWCNTs.

**Figure 13:**
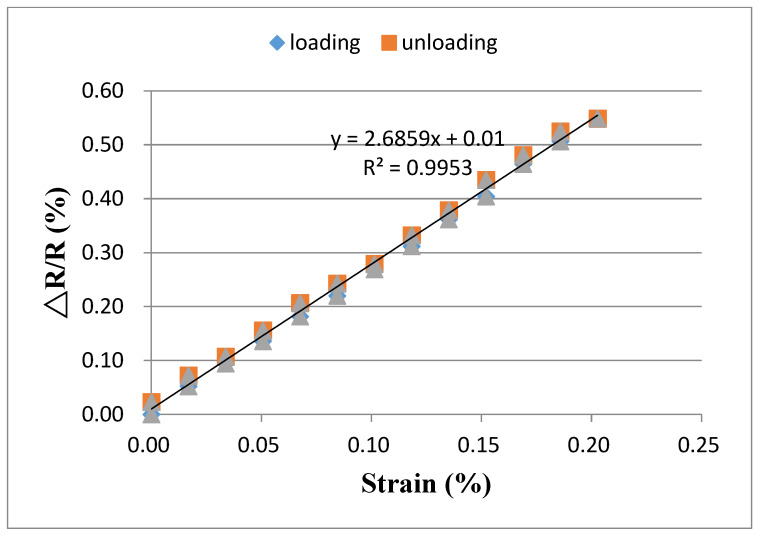
Relative resistance change vs. applied strain for the buckypaper made of low aspect ratio MWCNTs.

Figure 12 and Figure 13 show the relative resistance change in response to the applied strain for buckypapers made of high aspect ratio MWCNTs and low aspect ratio MWCNTs, respectively. It is clear that the relative resistance change exhibits a good linear relationship with the applied strain. By linearly fitting the relative resistance change and applied strain, the coefficient of determination achieves 0.99 for both buckypapers. The linearity and stability of the relative resistance change vs. applied strain demonstrate a great potential of the buckypaper as a strain sensor. The gauge factor of the buckypaper can be determined from the slope of the linear relationship. The gauge factors for the buckypapers consisted of high aspect ratio MWCNTs and low aspect ratio MWCNTs are 1.21 and 2.68, respectively. The resistance of the buckypaper can be characterized into two different types. One is the intrinsic resistance affected by the conducting or semiconducting type of MWCNTs, diameter and structural defects. The other is the contact resistance between MWCNTs controlled by the tunneling effect. It is well known that the intrinsic resistance of MWCNT is significantly lower than that of contact resistance. The buckypaper resistance is mainly dominated by the contact resistance between MWCNTs, while the intrinsic resistance has only a little effect on the buckypaper resistance. Thus, the piezoresistive response of the buckypaper is generally considered to be attributed to the change of the contact resistance. In the bending test, the buckypaper sensor adhered to the specimen was stretched, resulting in the increase of the distance between MWCNTs. The contact resistance of the buckypaper dominated by the tunneling effect between MWCNTs is increased due to the increase of the distance and reduction of the conductive paths. A positive piezoresistive behavior in response to the applied strain was found in the buckypaper sensor as shown in Figure 12 and Figure 13. The relative resistance changes of both buckypapers are linearly increasing with the increase of the applied strain. The difference in the piezoresistive response between the buckypapers composed of high aspect ratio MWCNTs and low aspect ratio MWCNTs can be attributed to the differences of the contact area and interaction between MWCNTs. The pore size and rope diameter of the buckypaper made of low aspect ratio MWCNTs are larger than that of the buckypaper composed of high aspect ratio MWCNTs as shown in Figure 5, leads to a larger contact area. Moreover, a layered structure of the buckypaper with high aspect ratio MWCNTs as shown in Figure 6a illustrates a good interconnection between MWCNTs, while a loose bound is found in the low aspect ratio MWCNT buckypaper as shown in Figure 6b. The distance between the low aspect ratio MWCNTs increased due to the tensile stretch is longer than that of high aspect ratio MWCNTs. Thus, the gauge factor of the buckypaper made of low aspect ratio MWCNTs is larger than that of high aspect ratio MWCNT buckypaper i.e., more sensitive to the applied strain.

### 3.5. Temperature Sensitivity

**Figure 14:**
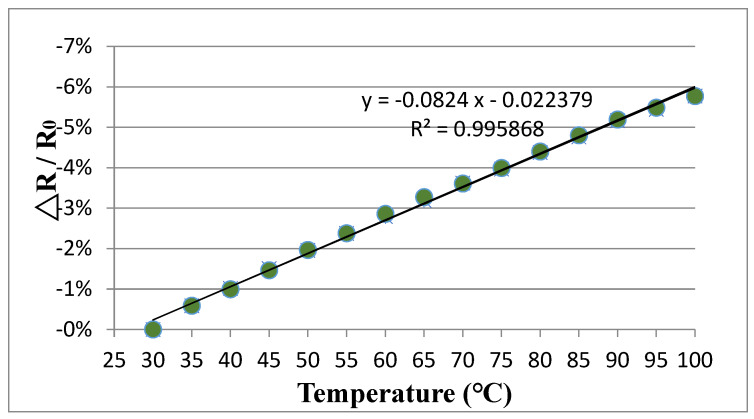
Relative resistance change vs. temperature change for the buckypaper made of high aspect ratio MWCNTs.

**Figure 15:**
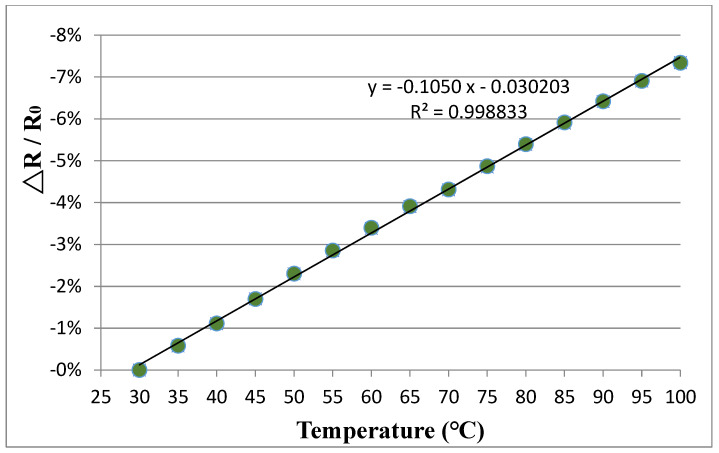
Relative resistance change vs. temperature change for the buckypaper made of low aspect ratio MWCNTs.

The fabricated buckypaper with dimensions of 30 mm × 10 mm rested on a glass substrate was placed in a vacuum oven and temperature varied from room temperature to 100 °C. Figure 14 and Figure 15 plot the relative resistance change of the buckypapers made of high aspect ratio MWCNTs and low aspect ratio MWCNTs in response to the temperature change, respectively. It can be seen that both buckypaper sensors exhibit a negative piezoresitivity, i.e., the resistance is decreasing with the increase of the temperature. As the temperature increases, the electron gains the thermal energy and transmits it to kinetic energy, leading to an increase of the mobility. The electron is more likely to leap across the interconnected MWCNTs instead of via tunneling. Thus, the resistance of the buckypaper decreases as the temperature increases. The relative resistance change of the buckypaper exhibits a good linear relationship with the temperature change as shown in Figure 14 and Figure 15. The temperature coefficient of resistance can be obtained from the slope of the resistance –temperature curve. The temperature coefficients of resistance for the buckypapers composed of high aspect ratio MWCNTs and low aspect ratio MWCNTs are $-8.24\times 10^{-2}$ °C^−1^ and $-1.05\times 10^{-1}$ °C^−1^, respectively.

## 4. Conclusions

Buckypapers were prepared by vacuum filtration method. The effect of MWCNT aspect ratio on the buckypaper performance was investigated in this work. The morphology of the buckypaper examined by field emission scanning electron microscopy depicts a self-assembled MWCNTs network due to the van der Waals forces. A layered structure and small pore size were found in the buckypaper made of high aspect ratio MWCNTs. This indicated a better interconnection between the high aspect ratio MWCNTs in comparison with the low aspect ratio MWCNTs. Mechanical properties of the buckypapers were characterized by the tensile and nanoindentation tests. Experimental results showed that the mechanical properties such as the Young’s modulus, tensile strength, fracture strain, and hardness of the buckypaper made of high aspect ratio MWCNTs were much higher than that of the buckypaper composed of low aspect ratio MWCNTs due to the better interconnection and smaller pore size. The piezoresistive behavior of the buckypaper was evaluated using a Keithley 2450 source meter. A positive piezoresistivity was found in response to the applied strain, while a negative piezoresistivity to the temperature change. The relative resistance change varied linearly with respect to both the applied strain and temperature change. The linearity and stability of the piezoresistive response demonstrate a great potential of the buckypaper as a strain and temperature sensor. Buckypaper consisting of low aspect ratio MWCNTs exhibits higher sensitivities in both strain and temperature compared with the buckypaper made of high aspect ratio MWCNTs.

## Figures and Tables

**Table 1:** Tensile properties of buckypapers composed of high aspect ratio MWCNTs and low aspect ratio MWCNTs.

Tensile Properties	High Aspect Ratio MWCNT Buckypaper	Low Aspect Ratio MWCNT Buckypaper
Young’s modulus GPa	0.679 ± 0.025	0.528 ± 0.019
Tensile strength MPa	17.3 ± 0.29	3.19 ± 0.10
Fracture strain	0.122 ± 0.003	0.018 ± 0.0005

Multi-walled carbon nanotubes (MWCNT).
